# Nanosheets of Metastable and Metamagnetic EuSe_2_

**DOI:** 10.1021/acs.chemmater.5c01424

**Published:** 2025-07-23

**Authors:** Salah Eddin El Jamal, Orlando C. Stewart, Tyler Hartman, Joel Swanson, Cheyenne Orozco, Sarah L. Stoll

**Affiliations:** Department of Chemistry, Georgetown University, 37th and O Streets NW, Washington, D.C. 20057, United States

## Abstract

We have identified solution conditions for synthesizing the metastable and metamagnetic semiconductor europium dichalcogenide, EuSe_2_, as two-dimensional nanosheets. We demonstrated phase control (EuSe or EuSe_2_) as well as shape control (blocks or nanosheets) and investigated the mechanism of nanosheet growth. The materials were characterized structurally using powder diffraction and microscopy (scanning electron microscopy, transmission electron microscopy, atomic force microscopy) as well as optical methods (UV–visible spectroscopy and Raman spectroscopy). The most interesting were the anisotropic magnetic nanosheet properties, both χ­(T) and M­(H). Using drop-cast films with high preferred orientation, it was possible to determine differences in the nanosheet magnetic properties both parallel and perpendicular to the plane of the nanosheet (along the *c*-axis). We observed eas*y*-axis antiferromagnetism along the *c*-axis and a Néel temperature close to that of the bulk. In addition, the critical field for the metamagnetic transition was reduced compared to the bulk.

## Graphical abstract

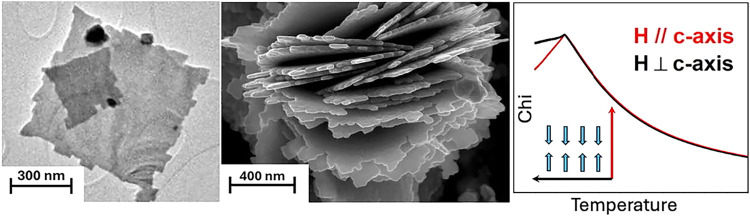

## Introduction

Interest in two-dimensional magnets has exploded due to recent discoveries such as room temperature ferromagnetism,^1–3^ layer-dependent magnetism,^4,5^ and thickness-dependent ordering temperatures.^6,7^ The search for new two-dimensional magnetic nanosheets has dominantly targeted van der Waals materials,^8^ where the strong within-layer interactions and weak between-layer bonding are thought to be important in identifying novel materials.^9,10^ In addition, van der Waals materials are key for 2D synthetic methods such as mechanical cleavage, sonication, and liquid or ion-assisted exfoliation.^11^ Unfortunately, there are limited examples of intrinsically ferromagnetic van der Waals materials. While there are examples of nonmagnetic materials that exhibit ferromagnetic order when grown as atomically thin layers on specific substrates,^12^ these provide no predictive guidelines. The ability to grow freestanding magnetic nanosheets unrestrained by these conditions would both broaden and deepen the understanding of magnetism confined to two dimensions.

We have been interested in the magnetic members of the layered lanthanide tri- and dichalcogenides. The trichalcogenides form only for tellurium, LnTe_3_, where the lanthanide (Ln = La, Ce, Pr, Nd, Sm, Gd, Tb, Ho, Er, Tm) is in the common trivalent oxidation state. The structure has a distorted rock-salt layer [Ln^3+^Te^2–^]^+^ and Te bilayers (with a van der Waals gap between Te layers).^13^ The LnTe_3_ materials are XY-type antiferromagnets with moments that couple within the plane, as typified by NdTe_3_.^14^ The absence of EuTe_3_ can be explained by the preferred divalent state of europium when it is bonded to the soft chalcogens. The second class, the dichalcogenide LnX_2_ (X = Se, Te), also has trivalent lanthanides, and the structure has distorted rock-salt layers of [Ln^3+^X^2–^]^+^ separated by single chalcogen layers [X_n_]^−^, as shown in Figure 1.^15,16^ In this case, the stability of Eu­(II) leads to an alternate structure-type for EuX_2_ (X = Se, Te), resulting in a more ionic material of alternating [Eu^2+^] and [X_2_^2–^] layers, isostructural with the alkaline-earth metal dichalcogenides (SrS_2_ and BaTe_2_).^17^ A comparison between the LnSe_2_ and EuX_2_ (X = Se, Te) structures in Figure 1 shows the respective layers of [LnSe]^+^ or [Eu^2+^] stacked along the *c*-axis. The europium dichalcogenides have anisotropic magnetism, with strong Ising-type in-plane ferromagnetic coupling and intralayer antiferromagnetism (A-type), with a field-dependent metamagnetic transition to ferromagnetism along the *c*-axis. This type of magnetic order is particularly appealing for investigating two-dimensional nanosheets, as the relative strength of the in-plane and out-of-plane coupling may be probed by thickness.

**Figure 1:**
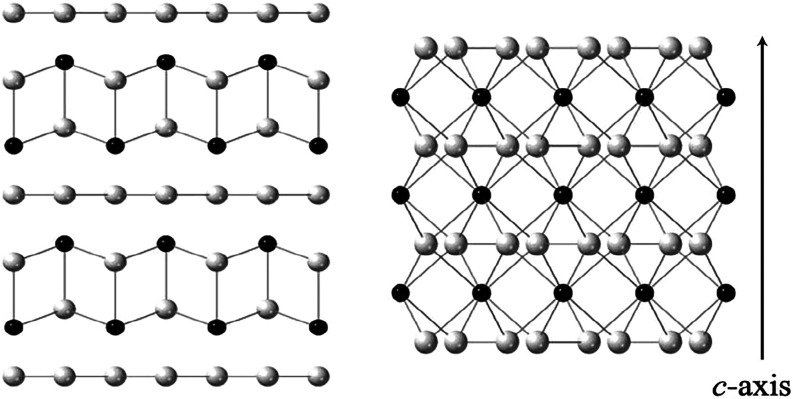
Structures for LnSe_2_ (left) and EuSe_2_ (right), where black spheres are the lanthanide (Ln or Eu) and the white ones are Se (or Te for Eu).

There are two highly motivating reasons to synthesize 2D magnetic nanosheets from solution: (1) colloidal synthesis is not limited to van der Waals materials, and (2) the higher yields allow use of a wider range of characterization tools, which is important for antiferromagnetic materials. There is growing interest in 2D antiferromagnetic materials for spintronic applications due to the reduced power consumption, absence of stray fields, and ultrafast dynamics.^18^ However, many of the techniques for studying 2D ferromagnets, which take advantage of a net moment (magneto-optical Kerr effect, magnetic circular dichroism, or, for metallic materials, the anomalous Hall effect), may not be useful probes for nonmetallic, spin-compensated antiferromagnets.^19,20^ Raman has emerged as an important tool for probing magnetism in 2D materials,^21^ but requires a specialized instrument with variable magnetic field and temperature capabilities. Alternatively, colloidal synthesis allows for detailed magnetic characterization using traditional magnetic susceptibility measurements that are useful for a wide range of magnetically ordered materials. Anisotropic growth of nanosheets with atomic-level thickness in colloidal conditions has been reported but remains an important challenge.^22^ Advances in the role of kinetics, ligand–surface interactions, and oriented attachment have provided some guidance for the mechanisms of anisotropic nanocrystal growth.^23^ There are examples of layered materials synthesized as colloidal monolayers,^24^ with controlled thickness,^25^ and a relatively unique approach for forming 2-dimensional nanosheets from nonlayered materials.^26,27^ Colloidal synthesis has the advantage that lower temperatures open the possibility to stabilize metastable materials.^28,29^ In addition, as the size is reduced, the relatively higher surface energy contribution to the lattice energy may favor a metastable form of the nanocrystal.^30^ As a result, known metastable polymorphs have been colloidally synthesized,^31,32^ and other nanocrystals have been discovered with new metastable structure-types not observed previously in the solid-state.^30^

We previously reported the solution synthesis of LnSe_2_ nanosheets from the single-source precursor, Ln­(Se_2_PPh_2_)_3_(CH_3_CN)_*x*_ (Ph = phenyl), and nanosheets (∼5 nm by 50–500 nm) were observed for Ln = La–Ho (but not Pm and Eu).^33^ In contrast, the precursor Eu­(Se_2_PPh_2_)_3_(CH_3_CN)_2_ formed EuSe nanocubes. Yan reported similar data for a more limited number of lanthanides using separate reagents (Ln­(acac)_3_, where acac = acetylacetonate and SeO_2_), for Ln = La–Nd, but found this phase competes with Ln_4_O_4_Se for Ln = Nd–Ho.^34^ This route stabilized EuSe_2_, but the morphology was distinct from the other LnSe_2_ in forming large nanobars (>100–350 nm). Recently, we discovered that by lowering the temperature and using separate reagents, (Eu­[N­(SiMe_3_)_2_]_3_ and [Et_2_NH_2_]­[Se_2_PPh_2_]), we were able to prepare phase pure EuSe_2_ as highly anisotropic nanosheets with lateral dimensions >500 nm and thicknesses of 25 (±4) nm (see Figure 2a). Modifying the solution amine from oleylamine (OLA) to hexadecylamine (HDA), the anisotropic growth could be further enhanced to form EuSe_2_ with lateral dimensions in the micron range, and thicknesses <14 (±2) nm (see Figure 2b). We have investigated the role of the temperature, reagent, and solvent on phase control as well as the mechanism of nanosheet formation. The EuSe_2_ nanosheets and ultrathin nanosheets were characterized structurally (X-ray powder diffraction, transmission electron microscopy, atomic force microscopy) and optically (UV–visible, Raman). What is most exciting is that using drop-cast films of ultrathin nanosheets, we could discern the magnetic anisotropy in the magnetic susceptibility measurements, both χ­(T) and M­(H).

**Figure 2:**
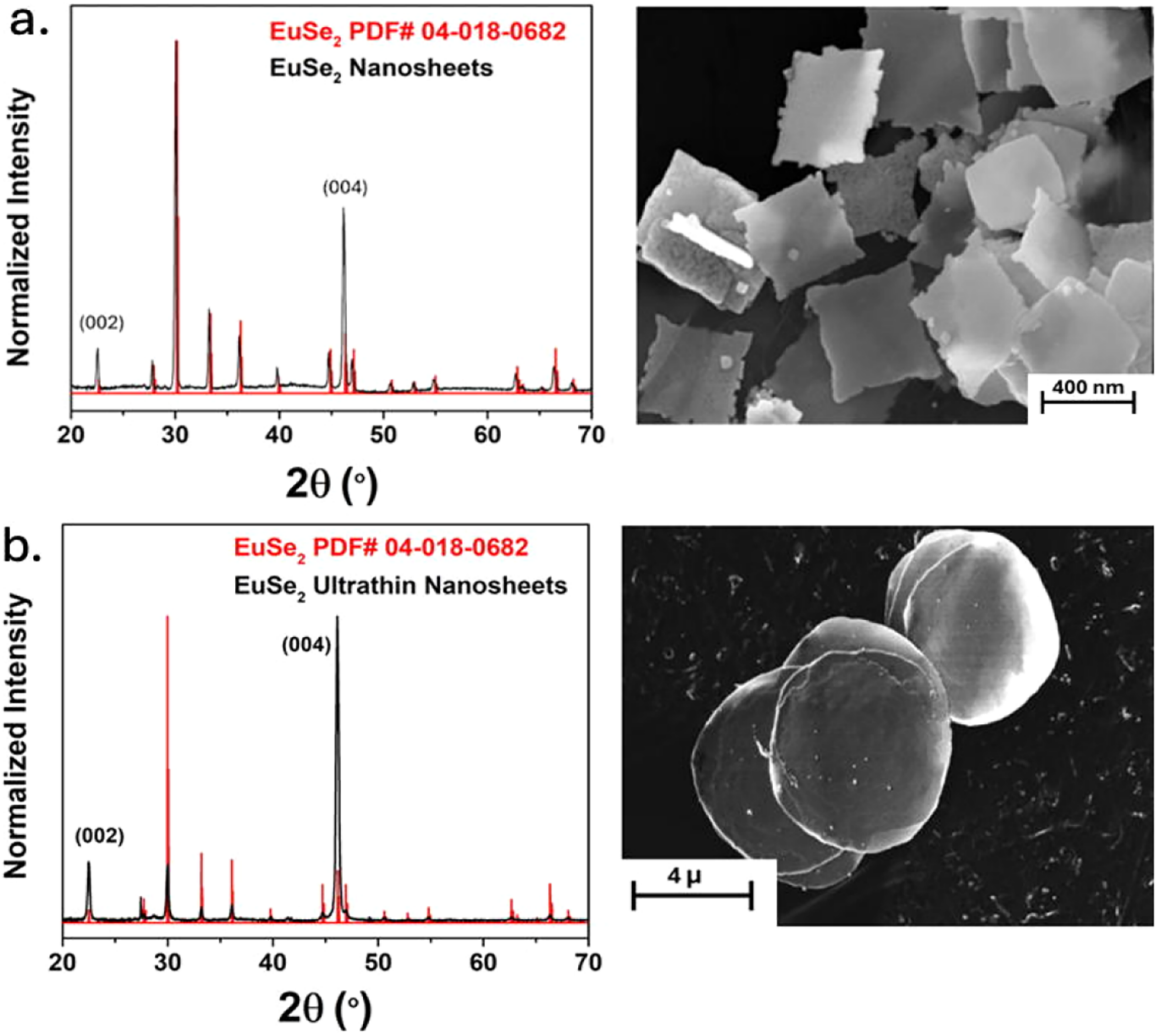
Powder X-ray diffraction (PXRD) and scanning electron microscopy (SEM) of (a) EuSe_2_ nanosheets from Oleylamine (OLA), (b) ultrathin nanosheets of EuSe_2_ in hexadecylamine (HDA).

## Results and Discussion

Europium monoselenide, EuSe, has been investigated intensely since the 1960s as a metamagnetic member of the magnetic semiconductors based on the europium monochalcogenides, EuX, where X = S, Se, or Te.^35^ It might seem surprising that europium diselenide was not discovered for another 30 years, but this simple binary material cannot be prepared by a direct combination of the elements at any temperature. Additionally, it thermally decomposes at 570 °C according to eq 1:

1 $${\mathrm{EuSe}}_{2}\left(s\right)\to \mathrm{EuSe}\left(s\right)+\mathrm{Se}\left(g\right)$$

However, using a molten alkali chalcogen flux, it was possible to synthesize crystals of the metastable EuSe_2_ at 750 °C.^36^ The metastability of EuSe_2_ is consistent with the calculated energy above the convex hull (EAH), which according to Materials Project is 0.014 eV/atom, a positive value leading to an assignment of “low stability”.^37^ By comparison, metastable nanoparticles (Co_*x*_Zn_1–*x*_S) have been reported with even larger EAH values.^38^

The reduced synthetic temperature of colloidal synthesis has many advantages for preparing metastable materials.^31^ What is interesting is the scant mention of EuSe_2_, given the number of reports of solution synthesis of EuSe nanoparticles. Syntheses of EuSe have been reported with a wide range of conditions, including various europium reagents (EuCl_3_, Eu­(NO_3_)_3_, Eu­(acetate)_3_), selenium reagents (Se dissolved in oleylamine, TOP-Se), and temperatures from 270° to 330 °C.^39–42^ Yet only Hasegawa mentions a weak impurity peak that could correspond to EuSe_2_.^43^ The first reported synthesis of EuSe_2_ nanoparticles (from Eu­(acac)_3_ and SeO_2_) was part of a broader effort to prepare two-dimensional nanosheets, and presumably the data was left in the Supporting Information due to the lack of anisotropic nanocrystal control and the large blocky nanocrystals that formed instead.^34^

### Phase Control

Generally, both the reactivity of the metal,^44^ as well as the reactivity of the anion, are important for phase control in nanoparticle synthesis.^45^ Metastable phases are generally stabilized at low temperatures with fast reacting precursors,^29^ while nanosheet growth is favored under slow chalcogen formation.^46^ In targeting EuSe_2_, we chose the highly soluble reagent with nitrogen donors, Eu­(HMDS)_3_ (HMDS = hexamethyldisilyzane, N­(Si­(CH_3_)_2_)_2_^–^), as the amide has been found to accelerate nanoparticle nucleation.^47^ Frequently, the metal halide and LiHMDS are used to form the reactive complex *in situ* (for example, in the synthesis of SnSe nanosheets);^25^ here, the preformed complex overcomes the low solubility of EuCl_3_ in oleylamine. The challenge was to identify a selenium reagent reactive enough to form the selenium-rich phase but at low enough temperatures to allow for anisotropic growth. Rather than the highly reactive (and toxic) SeO_2_, we used the salt of the diseleniumphosphinate (-Se_2_PPh_2_). By adopting low temperatures and using separate reagents for stoichiometric control, we were able to form high-aspect-ratio nanosheets of EuSe_2_.

The metastability of EuSe_2_ observed in the solid-state is evident in colloidal nanosheet syntheses by the low temperatures required (*T* < 280 °C). Temperature has a clear role in phase control; we observe pure EuSe at temperatures above 330 °C under the same conditions of reagent, concentration, and solvent used to form EuSe_2_. The phase identification as a function of the synthesis temperature is shown in Figure 3. It is worth noting that the concentration and reactivity of the chalcogen appear to be interrelated to the temperature of stability. The previously reported EuSe_2_ nanobars from Eu­(acac)_3_ and SeO_2_ were formed at 310 °C, a temperature that we find leads to EuSe with our reagents. This can be rationalized by the higher Se concentrations or the reactivity of SeO_2_, which shifts the equilibrium to EuSe_2_ at higher temperatures, as observed in the flux synthesis of the solid-state compound.

**Figure 3:**
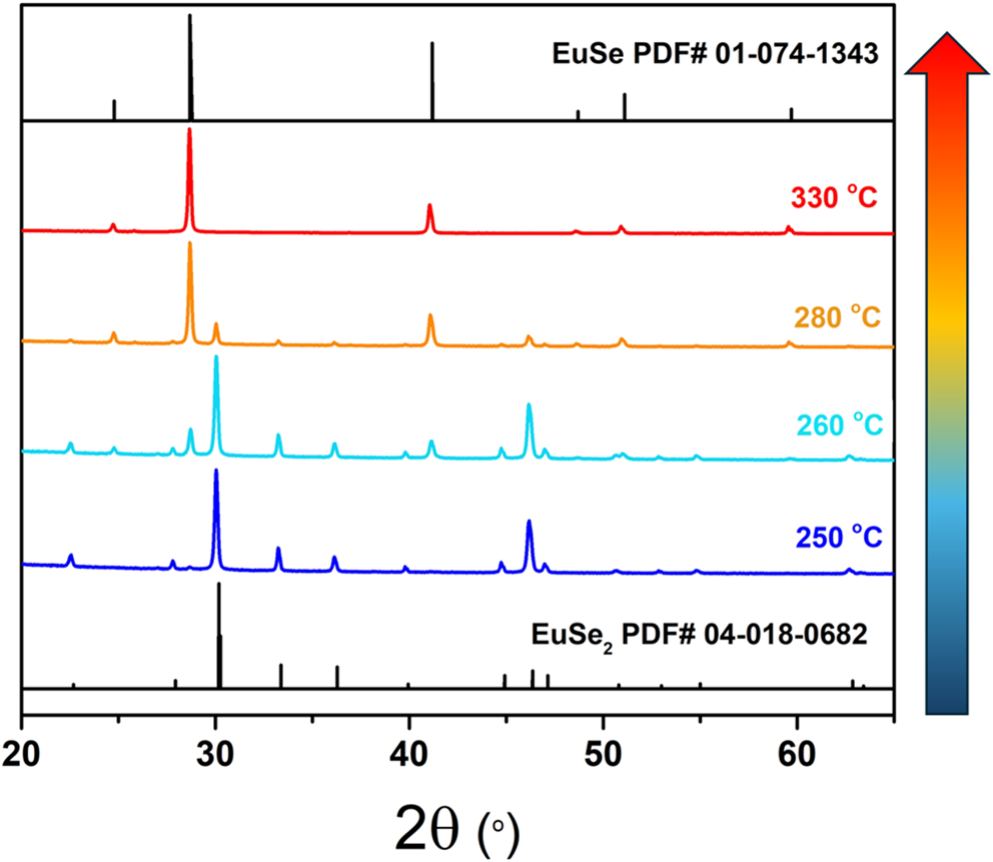
Phase formation (by PXRD) as a function of synthesis temperature. Single-phased EuSe_2_ stabilized at the lowest temperature.

To probe the temperature stability of EuSe_2_, we used preformed EuSe_2_ nanosheets and heated them in oleylamine (above 300 °C) and found the material converted to EuSe, analogous to the solid-state reaction in eq 1. We believe this is due to the decomposition of the EuSe_2_ rather than solvent extraction of Se, for several reasons. Solvent extraction of chalcogen to control phase has been demonstrated using trioctylphosphine to extract chalcogen from premade transition metal chalcogenides.^48^ However, while sulfur readily dissolves in oleylamine,^49^ selenium is considerably less soluble.^50^ In addition, the morphology of the nanosheet is lost; therefore, this conversion most likely involves the dissolution of EuSe_2_ and recrystallization of EuSe. Finally, thermal gravimetric analysis of EuSe_2_ nanosheets (under nitrogen) exhibited a broad decomposition between 420–520 °C (Figure S1), a temperature higher than used in our solution studies, and produced EuSe based on PXRD. Interestingly, it was not possible to stabilize EuSe_2_ by reacting preformed EuSe nanoparticles with an excess of Se at low temperatures. This suggests that EuSe_2_ nucleates in our low temperature synthesis, and it does not occur through an EuSe seed that converts by Le Chatelier’s principle due to the a high selenium concentration. We believe this provides further support that phase conversion involves dissolution and reformation. The low temperatures required for stabilizing EuSe_2_ may limit the rate of transformation from EuSe to EuSe_2_.

### Anisotropic Nanocrystal Growth

A preliminary criterion for predicting anisotropic nanocrystal growth is unequal bonding and reduced lattice symmetry.^26^ While van der Waals materials favor nanosheets when synthesized colloidally,^51^ they tend to rapidly stack to form multilayer materials.^22^ Mechanisms for anisotropic nanocrystal growth typically start with a consideration of what may be described as “facet-dependent growth”.^52^ Facet-dependent growth is where monomer addition along specific crystallographic faces leads to anisotropic materials from nanowires to two-dimensional nanosheets. A differential rate of growth along specific crystallographic directions is often governed by facet surface energy and surface dangling bonds.^27^ Small differences in surface free energy can be amplified by slowing nanocrystal facet growth to enhance anisotropic growth. The rate can be reduced through low temperatures, slow injection of precursors,^46^ the reactivity of reagents, or the use of facet-differentiating, coordinating ligands.^53^ We also consider other anisotropic nanocrystal mechanisms, including oriented attachment and screw-dislocation-driven anisotropy below.

### Dimensional Analysis of EuSe_2_ Nanosheets

In our prior studies of colloidal nanosheets, we have observed a range of nanosheet morphologies from folded wispy “wet-tissue” like materials (NdTe_3_),^14^ to sharply defined heavily stacked plates (SmSe_2_), to materials uniformly face up, with sharp corners and edges (e.g., HoSe_2_).^33^ The EuSe_2_ nanosheets had lateral dimensions that averaged from ∼700 nm to 1 μm based on TEM and were found to be single-crystalline, as shown in Figure 4a (histograms for TEM are in Figure S2, and elemental maps are in Figure S3). Generally, the materials appeared quite square, but nanosheets with rounded corners that approached a circular shape were found more frequently for nanosheets with larger lateral dimensions. Notably, in all cases, the edges of the nanomaterials were highly corrugated (additional TEM data in Figure S4). We used a combination of techniques to determine the nanosheet thickness. We considered the Scherrer analysis of PXRD as the most conservative and average ensemble thickness, and compared AFM for a sample of individual nanosheet thicknesses. Based on the line broadening of the (002) peak in PXRD (Figure S5), the Scherrer analysis resulted in a thickness of 25.6 nm (±4 nm). We have found that AFM measurements on a limited number of nanosheets typically lead to a lower range of thickness, and here found materials in the range 13.7 nm (±5.3 nm) from 52 nanosheets.

**Figure 4:**
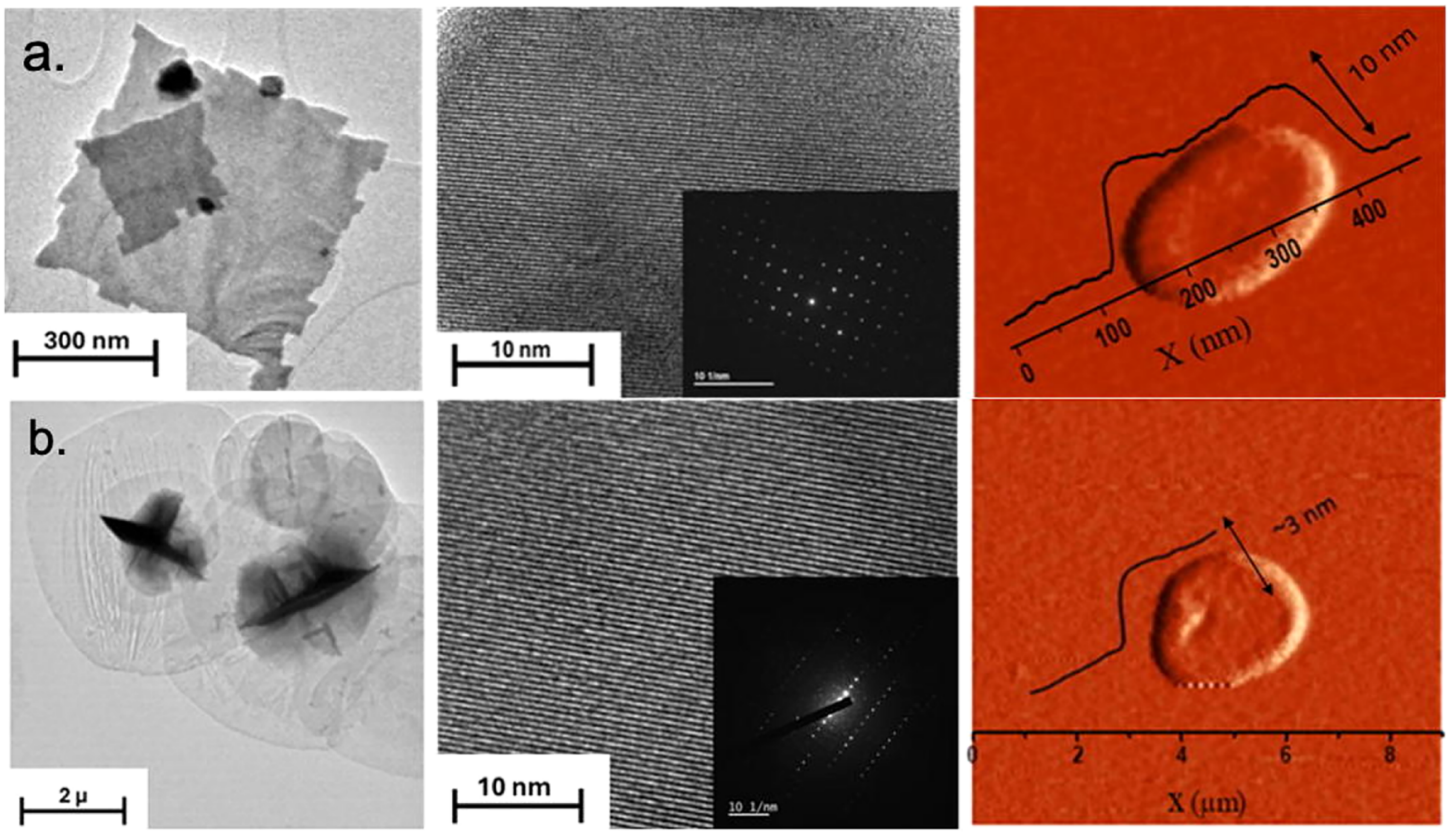
EuSe_2_ nanosheet from OLA (a) and ultrathin EuSe_2_ nanosheet from HDA (b). In each row, TEM (left), HRTEM with selected area electron diffraction inset (middle), and AFM (right).

One of the key methods for discerning the mechanism of nanocrystal growth is through direct microscopy imaging of nanocrystals isolated at multiple time intervals. One compelling piece of evidence for “oriented attachment” is that at the shortest time, the first nanocrystals isolated are recognizable as the repeat unit observed in larger aggregates. In the case of nanosheets, these are often described as “egg tray”-like structures.^54^ We have not isolated such a discrete nanocrystalline unit; however, our TEM images support a mechanism of edge attachment and coalescence under a range of conditions. For reactions halted after short periods of time (<5 min), we observe the initial fusing (recrystallization or coalescence) of thin box-like materials with a range of dimensions and very little organization (Figure S6). By 1 h, however, the electron diffraction patterns indicate the material is single-crystalline, with evidence of the edge addition of the box-like building unit (Figure 4a).

### Screw Dislocation Anisotropy

In addition to facet-dependent and oriented attachment growth, another mechanism to explain 2D nanocrystals is screw-dislocation-driven growth.^55^ Dislocation-driven two-dimensional growth has been observed in a range of nanomaterials, and models have been developed that show the “step velocity” can lead to either 1D or 2D growth.^56^ We have previously observed spiral crystal growth in the synthesis of two-dimensional nanosheets of SmSe_2_,^33^ and observed examples in EuSe_2_, as shown in Figure 5. This type of anisotropic crystal growth is the result of “defect-induced anisotropy”.^27,56^ In layered crystal growth, “monomers” (minimal building units) are integrated into the “kink” sites along the edges of the growing nanosheet.^57^ Screw dislocations create a line defect that forms a continuous step edge with a high density of sites for the monomer material to attach. As the growth extends the edge, it expands in a helical pattern, leading to spiral growth. Interestingly, with a small increase in the reaction temperature of 10 °C, the evidence of spiral growth is lost, but crystal growth of additional ad-layers along the face of the nanocrystal was captured by SEM in Figure 6. This shift from spiral growth to 2D nucleation and growth is associated with the extent of supersaturation.^57^ It is also notable that the addition of HDA to form the ultrathin EuSe_2_ appears to inhibit both spiral growth and this vertical layer-by-layer addition, *vide infra*.

**Figure 5:**
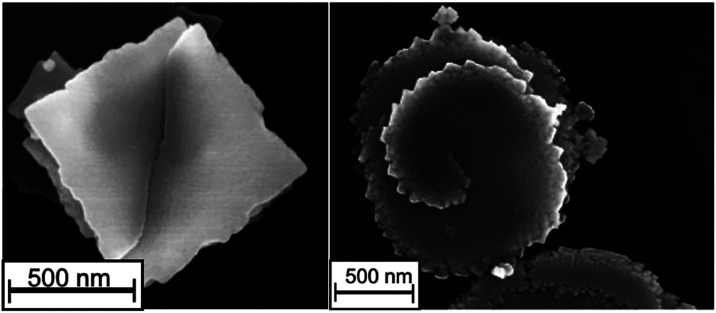
Evidence for screw dislocation in EuSe_2_ in scanning electron microscopy.

**Figure 6:**
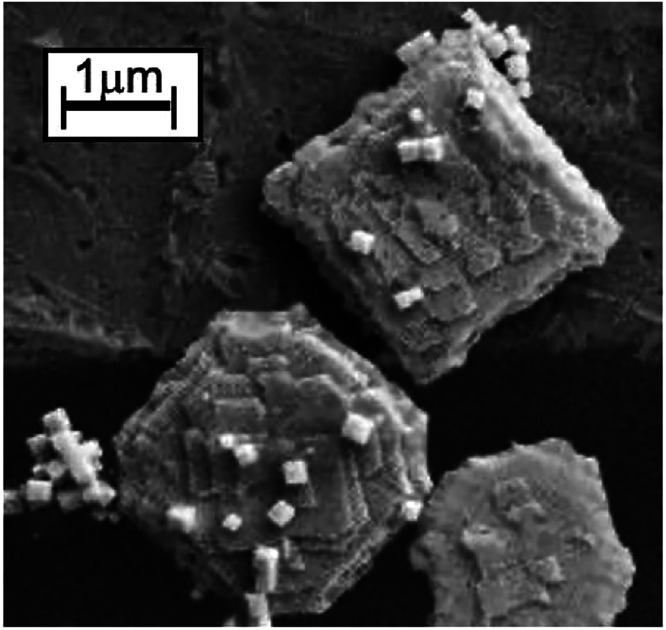
EuSe_2_ from Eu­(HMDS)_3_ in OLA at 260 °C from scanning electron microscopy.

### Ligand-Controlled Synthesis

The europium dichalcogenide nanosheet morphology is very sensitive to the metal chelate precursor as well as the solvent. We were able to synthesize EuSe_2_ from Eu­(oleate)_3_ and Eu­(acetate)_3_ to compare the phase and morphology with nanosheets from Eu­(HMDS)_3_. These reactions led to highly crystalline pure EuSe_2_ with no preferred orientation, based on PXRD (see Figures S7 and S8). The variation of europium precursor while keeping other aspects of the synthesis the same led to striking differences in nanocrystal shape and size (see Figure S9). Based on the data we have, our model is that the europium precursor is important for determining the rate of nucleation and monomer concentration (or reactivity of what forms the nanoparticles—the minimal building unit), while the solvent, which may play additional roles, caps the surface and influences the assembly and anisotropic nanocrystal growth.

If we define the rate of nanocrystal growth in terms of the nanoparticle volume per unit time of synthesis, then the EuSe_2_ from precursors Eu­(oleate)_3_ and Eu­(acetate)_3_ have greatly accelerated nanocrystal growth, compared to reactions using Eu­(HMDS)_3_. After 1 h, the average particle size of EuSe_2_ from these europium carboxylates is on the order of a few micrometers in all directions. The first reported EuSe_2_ “nanobars” (200–500 nm) were prepared from Eu­(acac)_3_, with comparable morphology to the materials from acetate or oleate here.^34^ The similarity in the europium reagent suggests a role for the carboxylate in enhancing the uncontrolled rapid growth (independent of facet). Chelates can affect growth by coordinating the metal or by coordinating the surface of the nanocrystal. Typically, facet-controlled growth is inferred by the presence of the ligand attached to the final nanomaterial, although these peaks can be weak in the FTIR. We measured the FTIR of all of the nanosheets, and the dominant peaks were from oleylamine, as expected for such a common solvent/capping ligand (Figure S10). We specifically looked for carboxylate stretches from the materials synthesized from Eu­(oleate)_3_ and Eu­(acetate)_3_, but only the latter exhibited weak peaks due to the presence of the carboxylate at 1548 cm^–1^ (asymmetric) and 1460 cm^–1^ (symmetric) (Figure S11). The difference in energy that suggests the carboxylate coordination mode has both oxygens bound to a single metal in an η^2^ fashion.^58^

All of the synthetic approaches testing the europium precursor used oleylamine as the solvent, so we initially ascribed the enhanced lateral growth observed for Eu­(HMDS)_3_ to the HMDS ligand. We infer that HMDS leads to a high concentration of small nanocrystals that edge attach and coalesce. Prior reports have found that HMDS acts as a ‘superbase’ that forms highly reactive metal amide intermediates, accelerating the nucleation process, leading to a high concentration of very small, initial nanocrystals.^48^ An analogous mechanism was reported in the synthesis of nanosheets of PbS, which was determined to occur via oriented attachment.^59^ Detailed TEM analysis of the growing PbS nanosheets found the small nanocrystal building block within the nanosheet in a close-packed-like structure, and the edge of the nanosheet exhibited a zigzag pattern of the exposed facets with the dimensions of the original nanocrystals. Importantly, the PbS nanosheets did not form unless small amounts of dichloroethane were added. The role of the chloride was to limit growth or stabilize the small nanocrystals, which then self-assemble into a monolayer, assisted by the coordination of the surfactant at the face of the nanosheet. Although we do not see a consistent small building block (Figure S6), we believe the corrugated edges visible in the TEM are due to the preferred edge attachment of square blocks.

### Solvent Effects

We modified our original synthesis by adding hexadecylamine (HDA) and found a striking difference in the extent of preferred orientation by PXRD and the nanosheet dimensions, as shown in the TEM (Figure 2b, TEM and histogram in Figure S12, elemental mapping in Figure S13). Surfactant-assisted anisotropic growth is usually thought to be due to surface binding and stabilization of specific crystallographic faces or through a “soft-template” effect.^54^ There is some limited evidence that small changes in steric hindrance and packing density of the solvent can make the difference between forming nanoparticles or nanosheets.^60^ While oleylamine and hexadecylamine are similar in sterics, they differ in chain length, and reported differences in facet-controlled nanocrystal growth have been observed.^61^ We believe the enhanced lateral growth of the EuSe_2_ nanosheets when hexadecylamine is added is due to the more efficient limiting of vertical growth and enhanced van der Waals interactions between the densely packed organic chains. It is also likely that the capping ligands control the assembly of the nanocrystals,^62^ as evidenced by the early-stage nanosheet formation observed by TEM (Figure S6).

### Dimensional Analysis of EuSe_2_ Ultrathin Nanosheets

Using TEM, the nanosheets from HDA were found to be quite translucent, micron-sized (>5.5 ± 0.45 μm), and more circular nanosheets, with less well-defined edges (Figure S12), compared to nanosheets synthesized using OLA (Figure 4a). To determine the bulk average thickness, the Scherrer calculation gave an average of 14.1 (±2) nm, which was much smaller than the 25.6 (±4) nm thickness for nanosheets from OLA. As expected, the ultrathin nanosheets (HDA) exhibited visibly greater line broadening of the 002 peak in the PXRD compared to nanosheets from OLA (Figure S5). Atomic force microscopy, while statistically more limited, provides evidence that the greatly increased lateral dimensions are accompanied by a correspondingly thinner nanosheet. The AFM data for the ultrathin nanosheets (HDA) showed a thickness of 4.66 ± 1.24 nm (from 32 nanosheets), which was measurably thinner than the AFM thickness of nanosheets from the OLA (13.7 ± 5.3 nm). In previous studies of colloidally grown nanosheets, we found that while the trend in thickness may be the same, the nanosheet thickness measurements by PXRD are generally larger than by TEM, which in turn are larger than by AFM.^14^

### Optical Properties

In the bulk, EuSe_2_ is a semiconductor with an indirect optical band gap of 1.42 eV,^36^ but our data does not cleanly match this value. However, we do see a distinction between the UV–visible absorption spectra of EuSe_2_ nanosheets (from the OLA and HDA) and EuSe_2_ nanoblocks (from europium carboxylates), reported in Figure S14. The EuSe_2_ nanosheets from OLA and the ultrathin (from HDA) nanosheets exhibit a shift in the optical absorption peak to shorter wavelengths (higher energy) relative to the EuSe_2_ nanoblocks. Whether assuming a direct Eg (nanosheet values of 1.62–1.73 eV versus nanoblock values of 1.55–1.58 eV) or indirect Eg (nanosheet values of 1.38 versus 1.35–1.37 eV), there was a clear shift. The Tauc plots are provided in the Supporting Information Figures S15 and S16. One of the interesting properties of 2D semiconductors is that, in many cases, the change in symmetry can lead to a change from indirect to direct band gap.^63^ A change from indirect to direct and blue shifting is consistent with size effects. However, defects and trap states due to ligand binding modes could lead to absorption edges at lower energy than the bulk.^64,65^ Our conclusions are tempered by the absence of luminescence in the nanomaterial and lack of temperature-dependent UV–visible absorption data, which would further elucidate the transitions.

### Raman Spectroscopy

In addition to confirming the identity of the capping ligand, the phonon structure of two-dimensional materials can be sensitive to nanosheet thickness.^66^ The Raman of bulk EuSe_2_ reported a single peak at 256 cm^–1^, corresponding to the Se–Se stretching mode.^36^ Our spectra are dominated by an intense peak at 259–262 cm^–1^, with a shoulder at 270 cm^–1^ (Figure S17). These are similar to the peaks found for the large EuSe_2_ formed from Eu­(oleate)_3_ and Eu­(acetate)_3_.

### Review of Magnetic Properties of Single-Crystal EuSe_2_ and EuTe_2_

Prior magnetic studies of single crystals of EuSe_2_ and EuTe_2_ are quite similar, supporting a model of highly anisotropic coupling. We summarize the literature in this section for comparison to our EuSe_2_ nanosheets in the next section. Studies of flux-grown single crystals of EuTe_2_ discovered magnetoresistance and colossal angular magnetoresistance,^67^ but EuSe_2_ has received much less attention. The reported magnetic data for EuX_2_ (X = Se, Te) are consistent with ferromagnetic coupling within the layer (*ab* plane), eas*y*-axis antiferromagnetic coupling between layers (along the *c*-axis), and a metamagnetic transition to ferromagnetism.^68^ While broadly similar, there are subtle differences in the magnetic data, perhaps due to differences in the strength of the magneto-crystalline anisotropy.

Reports of anisotropy in the magnetic structure of EuSe_2_ and EuTe_2_ used crystallographically oriented single crystals in an applied field.^17,66^ Based on the χ­(T) of single crystals with the applied field parallel to the *c*-axis (*H*_∥c_), antiferromagnetism is confirmed by the strong Néel peak. Because the easy axis is along c, when the field is perpendicular to the *c*-axis (*H*_⊥c_), rather than a drop in susceptibility below the Néel temperature, the susceptibility becomes constant as the antiparallel moments are forced off the *c*-axis. Similarly, for a comparison of isothermal M­(H) measurements when the field is parallel to c (*H*_∥c_), both materials exhibit small moments at low fields due to the cancellation of the moments in adjacent layers. Correspondingly, when the field is perpendicular to the *c*-axis (*H*_⊥c_), both materials exhibit larger moments at low fields and require much higher fields to saturate. However, in the case of EuTe_2_, there is a clear spin-flop transition at 3T when *H*_∥c_. By contrast, EuSe_2_ has a poorly understood small inflection at 1 mT and a sharper metamagnetic transition at 1.5 T.

### Magnetism of EuSe_2_ Ultrathin Nanosheets (Powders)

Our measurements of the χ­(T) of the ultrathin EuSe_2_ nanosheet powders (Figure S18) confirm antiferromagnetism, with a Néel temperature close to that measured for bulk EuSe_2_, 8 (±0.5) K (measured at 0.1 T). An analysis of the 1/χ data had a slight curvature, which had little effect on the moment but some variation in the Weiss constant, depending on the temperature range of the fit. Using data from 100 to 300 K, we determined a moment of μ_eff_ ∼7.9 ± 0.2 μ_B_ (Bohr Magnetons), where the spin-only expected value is 7.9 μ_B_ for divalent europium. The Weiss constant or Θ for the ultrathin material was ∼5 K, reduced from the positive Θ∼13–15 K in the bulk. In the Supporting Information, we also include the χ­(T) and M­(H) data for the EuSe_2_ nanosheets from OLA (Figure S19), as well as the Curie–Weiss fits for micron-sized EuSe_2_ materials from Eu­(oleate)_3_ and Eu­(acetate)_3_ (Figure S20). In all cases, the μ_eff_ ranged between 7.3 and 7.9 μ_B_, but the Weiss constant values ranged from Θ ∼−1.4 to 5.7 K. Consistent with the large moment, the M­(H) data indicate a saturation magnetization of *M*_s_ ∼33,000 emu/mol, close to that of the bulk (39,000 emu/mol or compared to *M*_s_ = gSN_A_μ_B_ where *g* = 2, *S* = 7/2). Similar to the reported bulk, there is a small inflection at low fields and a metamagnetic transition at ∼1 T (based on the derivative of the M­(H)).

### Anisotropic Magnetic Measurements of EuSe_2_ Ultrathin Nanosheet Films

As described for single crystals of EuSe_2_, there are distinct differences in both χ­(T) and M­(H) measurements depending on whether the field is parallel or perpendicular to the *c*-axis. Due to the high preferred orientation of the ultrathin EuSe_2_ nanosheets synthesized from HDA, we were able to measure oriented nanosheets by preparing drop-cast thin films of ultrathin EuSe_2_ (see Figure S21). Electron diffraction of the nanosheets confirms that the *c*-axis is perpendicular to the plane of the nanosheets. By lying flat “face up” on the substrate, it was possible to use PXRD to confirm that the nanosheets were oriented with the *c*-axis perpendicular to the film. In Figure 7, the magnetic measurements, χ/χ_(TN)_ vs T at 0.1 T, are shown for fields *H*_∥c_ and *H*_⊥c_ to the *c*-axis of EuSe_2_ nanosheet films. We find that measurements (*H*_∥c_ and *H*_⊥c_) are quite similar as the temperature decreases but deviate below the Néel temperature (T_N_). At T_N_, the measurement with *H*_∥c_ exhibits a sharp decrease in the magnetization as expected for AF transition, while the measurement with *H*_⊥c_ exhibits a moment that is relatively constant below the T_N_. This deviation is characteristic of a material with an easy antiferromagnetic axis along the c-direction.

**Figure 7:**
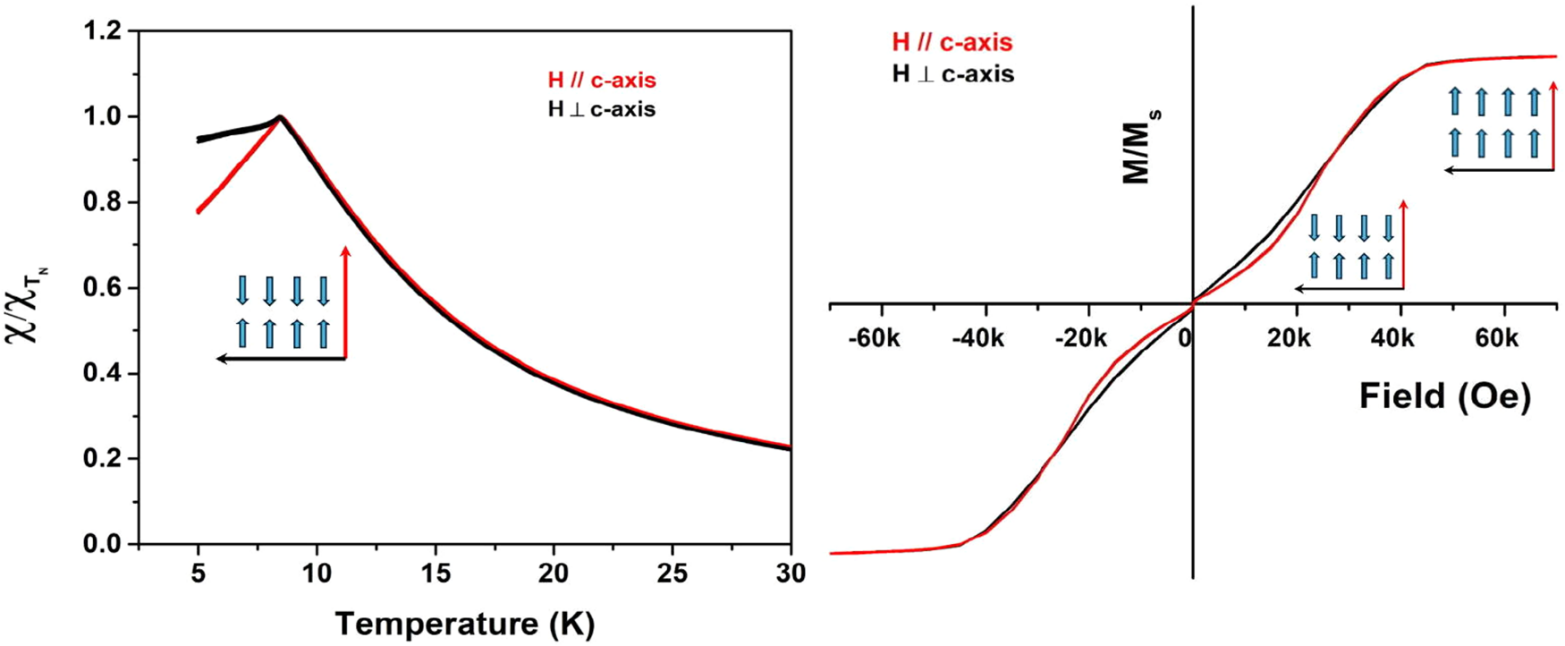
Magnetic data on oriented films of ultrathin EuSe_2_: normalized χ­(T) parallel and perpendicular to the easy axis (c) (left), and M/M_s_(H) at 2.5 K parallel and perpendicular to the easy axis (c) (right).

We were also able to measure the M­(H) data with fields *H*_∥c_ and *H*_⊥c_ (at 2.5 K). As observed in single-crystal data, the M/M_s_ as a function of applied field, the magnetization with the field *H*_∥c_ is smaller than the magnetization measured with the field *H*_⊥c_ at low fields. There is also a slight deviation as the moments approach saturation, and as seen for single-crystal data, where the moment with *H*_∥c_ saturates at a slightly lower field when compared to when *H*_⊥c_. Although this effect is small, it is reproducible for many samples. Based on the derivative of the M/M_s_(H), the critical field for the metamagnetic transition is 1 T compared to 1.5 T in the bulk. A lower critical field suggests greater stability of the ferromagnetic phase due to reduced dimensions.

## Conclusions

We have discovered a solution synthesis to control both the phase and the morphology of metastable EuSe_2_ nanosheets. Colloidal synthesis of nanosheets is likely to accelerate the mapping of anisotropic magnetic materials with potential spintronic applications by providing a low-cost method for making macroscopic amounts of nanosheets for rapid structure–property relationships. One of the significant achievements of this work is that we were able to elucidate the anisotropic properties of nanosheets using thin nanosheet films, due to the highly preferred orientation of the ultrathin nanosheets. Traditional methods for the synthesis of atomically thin nanosheets have a relatively high barrier for the formation of this metastable material, and the methods that are successful for measuring ferromagnetism in 2D materials would not work for a magnetically complex system such as EuSe_2_. Additionally, we believe that ensemble magnetic measurements have a statistical advantage over single nanosheet measurements, in that subtle differences due to atomic-level changes in surface chemistry are averaged out, thereby reducing artifacts of the measurement conditions, measurement bias, and providing more representative data.

## Experimental Section

### Materials

Eu­(III) trifloromethanesulfonate (98%, Sigma), Eu­(III) nitrate hexahydrate (99.9%, Strem Chemicals), lithium hexamethyldisilyzane (or lithium bis­(trimethylsilyl)­amide) (97%, Sigma), hexadecylamine (98%, Sigma), selenium (99.9%, Sigma), sodium oleate (>97%, Tokyo Chemical Industry), ethanol (>99.5%, anhydrous, Sigma), diethylamine (>99.5%, Sigma) diphenylphosphine (98%, Sigma), diethyl ether (>99.7%, anhydrous, Sigma), tetrahydrofuran (>99.9%, anhydrous, Sigma), and hexanes (>98%, anhydrous, Sigma) were used. Eu­(III) acetate hydrate (99.9%, Strem Chemicals) was degassed at 150 °C for 5 h before storing it in the glovebox. Oleylamine (98%, Sigma) was distilled under vacuum to a clear and colorless liquid and then stored in the glovebox.

### Eu­(HMDS)_3_ Synthesis

In a glovebox, LiN­[Si­(CH_3_)_3_]_2_ (2.55 g, 15 mmol) was mixed with THF (30 mL) and hexanes (30 mL) in a Schlenk flask. While the mixture was being stirred, Eu­(CF_3_SO_3_)_3_ (3 g, 5 mmol) was added to the reaction mixture. The flask was transferred to the Schlenk line and kept refluxing at 70 °C for 3 h under argon. The mixture was then vacuumed off until a dry orange precipitate was obtained. The product was transferred into the glovebox, vacuum filtered, and washed with hexanes (15 mL) and toluene (15 mL). The solvents were then vacuumed off, and the orange powder was stored in a nitrogen glovebox.

### Eu­(oleate)_3_ Synthesis

Eu­(NO_3_)_3_·6H_2_O (2.24 g, 5 mmol) and sodium oleate (4.55 g, 15 mmol) were added to a mixture of deionized water (15 mL), ethanol (10 mL), and hexanes (7 mL) and heated to reflux. The mixture was refluxed for 4 h while stirring. Then, the mixture was immediately transferred to a separatory funnel, where two phases were formed, a yellow organic layer over a transparent aqueous layer. The translucent organic layer was then washed in 4 cycles of 40 mL of deionized water. The organic layer was then transferred into a Schlenk flask and degassed at 120 °C for 2 h, and a translucent yellow solid product was obtained. The product was then stored in the glovebox.

### Synthesis of Diethylammonium Diselenodiphenylphosphinate [Et_2_NH_2_] [PPh_2_Se_2_]

In a glovebox, Se (3.6 g, 46 mmol) with 4 mL of diphenylphosphine was dissolved in ethanol (40 mL) in a Schlenk flask. Then, the flask was transferred to a Schlenk line and heated at 70 °C under argon. Diethylamine (3 mL) was then hot injected into the reaction mixture. The reaction mixture was cooled to room temperature. After that, the Schlenk flask was placed in the freezer overnight to allow recrystallization of the product. The next day, a white precipitate formed and was vacuum filtered and washed with diethyl ether (40 mL). The yield was 71%, and the collected white powder was degassed and placed in the glovebox.

### Synthesis of Europium Diselenide Nanosheets and Ultrathin Nanosheets

In a nitrogen glovebox, Eu­(HMDS)_3_ (44 mg, 0.07 mmol), [Et_2_NH_2_]­[PPh_2_Se_2_] (180 mg, 0.4 mmol), and OLA (6 mL) were added, in order, to a round-bottom flask and fitted with a condenser and a gas adapter. The setup was then transferred to a Schlenk line and degassed at 120 °C for 20 min and then heated at 250 °C for 1 h under argon. The reaction mixture was allowed to cool down to room temperature. The product was washed with hexanes and ethanol in 3 cycles. The identity of the product was confirmed by PXRD. For ultrathin nanosheets, the synthesis was carried out as described previously for EuSe_2_ nanosheets but with the addition of hexadecylamine (250 mg, 1 mmol).

### Synthesis of EuSe_2_ Large Nanoblocks

Eu­(acetate)_3_ (23 mg, 0.07 mmol), [Et_2_NH_2_]­[PPh_2_Se_2_] (180 mg, 0.4 mmol), and OLA (6 mL) were added, in order, to a round-bottom flask and fitted with a condenser and a gas adapter. The reaction mixture was degassed at 120 °C for 20 min on a Schlenk line. The reaction was then heated at 250 °C under argon for 1 h, and a dark brown precipitate formed. The reaction mixture was allowed to cool to room temperature, and the product was washed with hexanes and ethanol in 3 cycles and stored in hexanes. The synthesis of EuSe_2_ nanoblocks from Eu­(oleate)_3_ was done following the same protocol but with Eu­(oleate)_3_ (70 mg, 0.07 mmol) instead of Eu­(acetate)_3_.

### Characterization

X-ray powder diffraction patterns were obtained using a Rigaku Ultima IV X-ray powder diffractometer with Cu Kα radiation at 40 kV and 30 mA and a D/teX silicon strip detector. Samples were prepared for SEM and TEM measurements by drop-casting dilute nanomaterial solutions in hexanes on carbon-coated copper TEM grids. Glass slides were used for Raman, and silicon wafers were used for AFM. High-resolution TEM (HRTEM) and elemental mapping analyses were carried out on a JEOL JEM-2100F FEGTEM instrument operated at 200 kV at the Advanced Imaging and Microscopy Lab at the University of Maryland. Scanning electron microscopy (SEM) images were taken with a Zeiss SUPRA 55-VP scanning electron microscope at an acceleration voltage of 20 kV with an in-lens detector. UV–vis diffuse-reflectance spectra of powder samples were obtained by mixing the powder with MgO using a Cary 5000 UV–vis-NIR spectrometer. Thermogravimetric Analysis was performed on an SDT650 TA Instrument. The sample, 3–5 mg, was heated in a ceramic pan under nitrogen (50 mL/min) from 20 to 700 °C, with a heating rate of 5 °C/min.

FTIR measurements were performed on an Agilent Cary 630 FTIR instrument equipped with a diamond ATR accessory. Spectra were acquired with 16 scans and a resolution of 2 and analyzed using MicroLab software from Agilent. FTIR samples were prepared by drop casting a concentrated solution of nanoparticles dissolved in hexanes.

AFM samples were prepared by drop casting a dilute solution of nanoparticles dissolved in hexanes onto a silicon wafer. The surface topography was acquired with an NTEGRA scanning probe microscope (NT-MDT) operated in semicontact/tapping mode. The probe is made from single-crystal silicon with a nominal spring constant of ∼12 N/m. Raman samples were prepared by drop-casting a solution of nanoparticles dissolved in hexanes on a glass slide. Measurements were performed on a Horiba Raman microscope equipped with a 532 nm laser. The instrument was interfaced with an Olympus BH2-UMA optical microscope, and a magnification factor of 100× was used. Spectra were recorded in extended scan mode from 50 to 1000 cm^–1^ and analyzed using the WiRE 2.0 software package.

Magnetic susceptibility was obtained on a Quantum Design MPMS3 SQUID magnetometer. Data were collected using a temperature sweeping mode from 2.5 to 300 K at 1000 (0.1 T) and 30,000 Oe (3T) under both zero-field-cooled (ZFC) and field-cooled warming (FCW) conditions. The Curie–Weiss analysis was done on FC data at 30,000 Oe (3T) between 100 and 300 K. Magnetic hysteresis data was collected at 2.5 K from −7 to 7 T. All data were corrected for diamagnetic contributions using Pascal’s constants.

## Supplementary Material



## Notes

Additional information on the magnetic measurements, analysis, and anisotropic magnetic measurements. Additional data include TGA of EuSe_2_ nanosheets and PXRD (Figure S1), the TEM and histogram of EuSe_2_ nanosheets (Figure S2), elemental mapping of EuSe_2_ nanosheets (Figure S3), TEM of EuSe_2_ nanosheet edges (Figure S4), a comparison of 002 PXRD peak for different syntheses (Figure S5), additional TEM of EuSe_2_ nanosheets over time (Figure S6), PXRD of synthesis with Eu­(acetate)_3_ and Eu­(oleate)_3_ (Figures S7 and S8), and SEM comparing morphology of large EuSe_2_ (from oleate and acetate) (Figure S9). The FTIR of EuSe_2_ nanosheets from OLA and HDA (Figure S10), FTIR of nanoblocks of EuSe_2_ from oleate and acetate in the carboxylate region (Figure S11), TEM of ultrathin EuSe_2_ and histogram (Figure S12), and elemental mapping of ultrathin EuSe_2_ nanosheets (Figure S13). The UV–visible absorption spectra comparing EuSe_2_ from all syntheses (Figure S14), Tauc plot for direct band gap (Figure S15), Tauc plot assuming indirect band gap (Figure S16), and Raman spectroscopy of EuSe_2_ nanosheets (Figure S17). Magnetic data, including χ­(T), M­(H), and Curie–Weiss fit for EuSe_2_ ultrathin nanosheets (Figure S18), and magnetic data, including χ­(T), M­(H), and Curie–Weiss fit for EuSe_2_ nanosheets from OLA (Figure S19). Curie–Weiss fits for the EuSe_2_ from Eu­(oleate)_3_ and Eu­(acetate)_3_ (Figure S20), and PXRD of EuSe_2_ ultrathin nanosheets on Si wafer for magnetic measurements (Figure S21) (PDF)
