# Supplementary material for: Nanosheets of Metastable and Metamagnetic EuSe2
Source: Chem Mater. 2025 Jul 23;37(15):6048–58. doi: 10.1021/acs.chemmater.5c01424 (PMC12355645; doi:10.1021/acs.chemmater.5c01424)
Supplement: Supplementary file 1 [file cm5c01424_si_001.pdf]

Supporting Information for Nanosheets of Metastable and Metamagnetic $EuSe_2$

Salah Eddin El Jamal[1], Orlando C. Stewart Jr.[1], Tyler Hartman[1], Joel Swanson[1], Cheyenne Orozco[1], and Sarah L. Stoll*[1]

[1]Department of Chemistry, Georgetown University, 37th and O Streets NW, Washington, D.C. 20057, United States

E-mail: sls55@georgetown.edu

S1. Thermogravimetric analysis (TGA) of $EuSe_2$ under nitrogen and PXRD post-heating.
S2. SEM of $EuSe_2$ nanosheets, and histogram from TEM data.
S3. Elemental mapping of $EuSe_2$ nanosheets.
S4. TEM of edges of $EuSe_2$ nanosheets, evidence of edge attachment and coalescence.
S5. Comparison of 002 peak for all 4 syntheses and thickness histograms of $EuSe_2$ nanosheets.
S6. TEM evidence for Oriented Attachment after 5 mins and 1 hour.
S7. X-ray Powder Diffraction Pattern of $EuSe_2$ from $Eu(acetate)_3$.
S8. X-ray Powder Diffraction Pattern of $EuSe_2$ from $Eu(oleate)_3$.
S9. SEM comparison of nanocrystal morphology from $Eu(acetate)_3$ and $Eu(oleate)_3$.
S10. FTIR of the $EuSe_2$ nanosheet and ultra-thin $EuSe_2$.
S11. FTIR of the large $EuSe_2$ (from oleate and acetate) in the carboxylate region.
S12. TEM of ultra-thin $EuSe_2$ (from HDA), and histogram.
S13. Elemental mapping of ultra-thin $EuSe_2$ nanosheets.
S14. UV-visible absorption spectroscopy of Thin $EuSe_2$, Ultra-thin $EuSe_2$ and $EuSe_2$ from acetate and oleate.
S15. Tauc Plots of Thin $EuSe_2$, Ultra-thin $EuSe_2$ and $EuSe_2$ from acetate and oleate, direct Eg.
S16. Tauc Plots of Thin $EuSe_2$, Ultra-thin $EuSe_2$ and $EuSe_2$ from acetate and oleate, indirect Eg.
S17. Raman of $EuSe_2$ nanosheets.
S18. Magnetic data of $EuSe_2$ ultra-thin nanosheets (HDA).
S19. Magnetic data of $EuSe_2$ Nanosheets from OLA
S20. Curie-Weiss Plot of the $EuSe_2$ from $Eu(acetate)_3$ and $Eu(oleate)_3$.
S21. PXRD of $EuSe_2$ ultrathin nanosheets on Si wafer for thin film magnetic measurements.

Magnetic Measurements.

Samples for magnetic measurements were prepared in a vibrating sample magnetometry (VSM) sample powder holder. Magnetic measurements were done using superconducting quantum interference device (SQUID) on a Quantum Design Magnetic Property Measurement System (MPMS3) system. Temperature sweeping mode from 2.5 to 300 K of an applied field of 1000 Oe (0.1 T) was used to collect data under zero-field cooled (ZFC) and field-cooled (FC) conditions. The Curie−Weiss analysis was done on ZFC data at 1000 Oe (0.1 T) between 100 and 300 K. Collected data was corrected for diamagnetic contributions using Pascal's constants.[1] Magnetic hysteresis data was collected at 2.5 K from -7 to 7 T.

Preparation of silicon wafer.

Ultrathin $EuSe_2$ nanosheets were dispersed in hexanes and then the solution was drop cast on a silicon wafer to form a thin layer of the material. The silicon wafer was then secured on a quartz paddle sample holder for ⊥ to c-axis measurement and a standard sample holder for ∥ to c-axis measurement. MPMS3 sample geometry simulator software from QUANTUM DESIGN was used to correct geometry effects of magnetic moment measurements in the QUANTUM DESIGN MPMS3 magnetometer.

Information about how the data is collected, processed, and analyzed. Particularly for the in-plane and out of plane data.

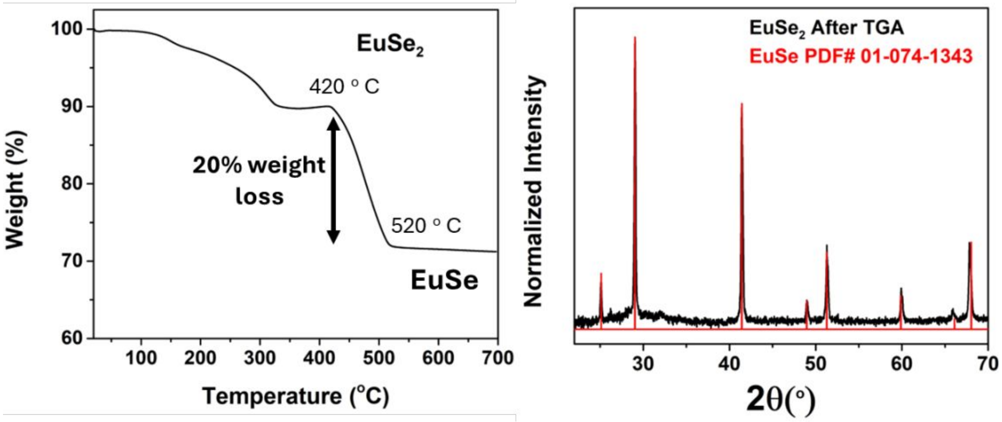

S1. Thermogravimetric analysis (TGA) of $EuSe_2$ under nitrogen (left), PXRD of material from TGA post heating (right). Calculated mass loss should be 25% for $EuSe_2$ to form EuSe.

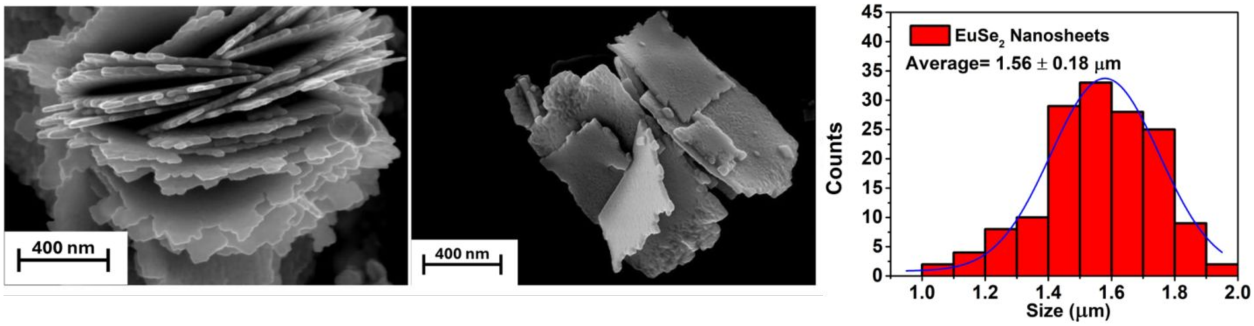

S2. SEM of $EuSe_2$ nanosheets, and histogram from TEM data (from 150 nanosheets).

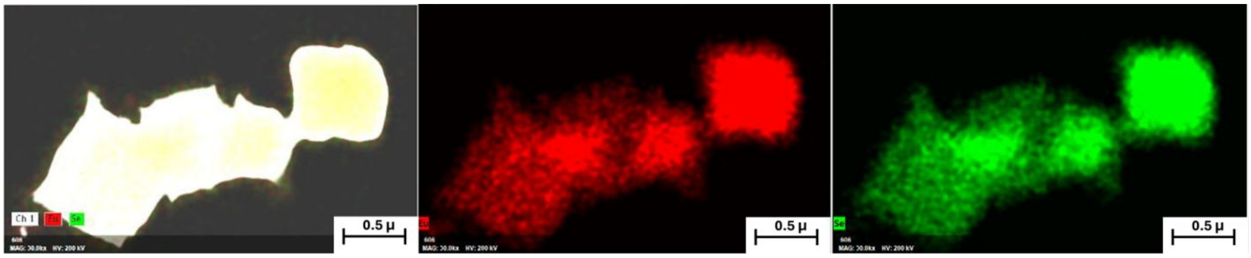

S3. Elemental mapping of $EuSe_2$ nanosheets.

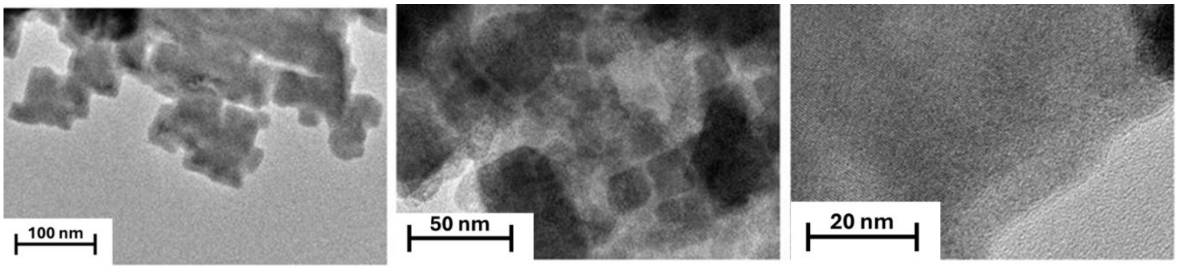

S4. TEM of edges of $EuSe_2$ nanosheets, evidence of attachment and coalescence.

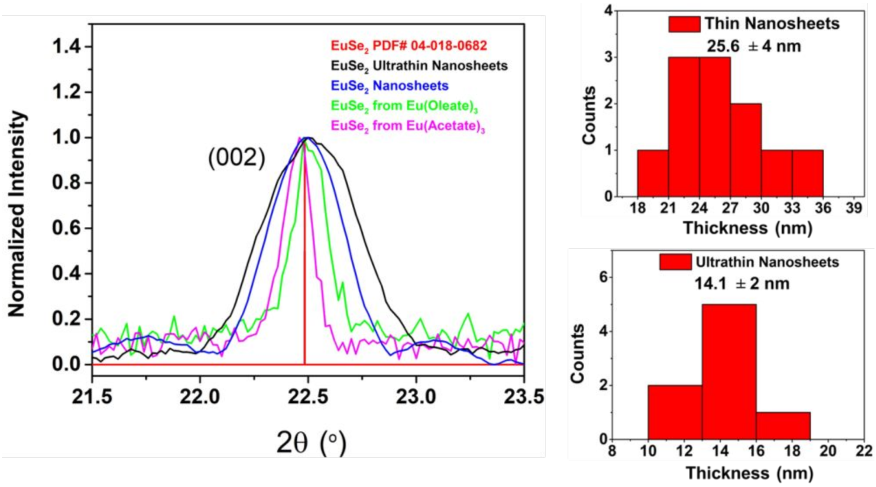

S5. Comparison of *002* peak for 4 syntheses: thin nanosheets of $EuSe_2$ (OLA, blue, 22nm thick), ultra-thin nanosheets (HDA, black, 14 nm thick), micron sized $EuSe_2$ from $Eu(O_2CCH_3)_3$ and $Eu(oleate)_3$ in pink and green respectively.

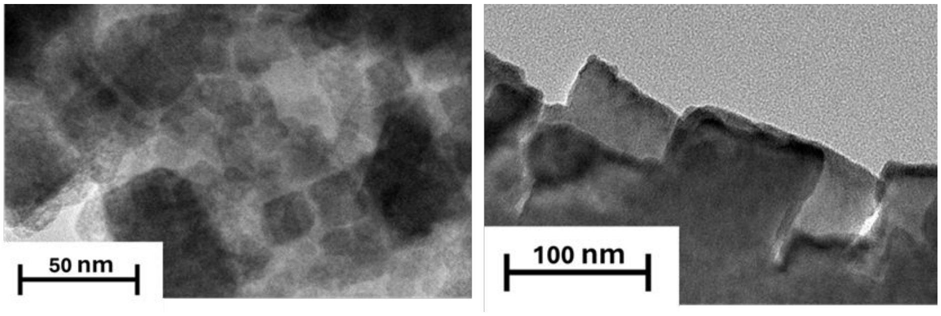

S6. TEM evidence for Oriented Attachment, nanosheets formed after 5 minutes (left) edges of large nanosheets after 1 hour (right).

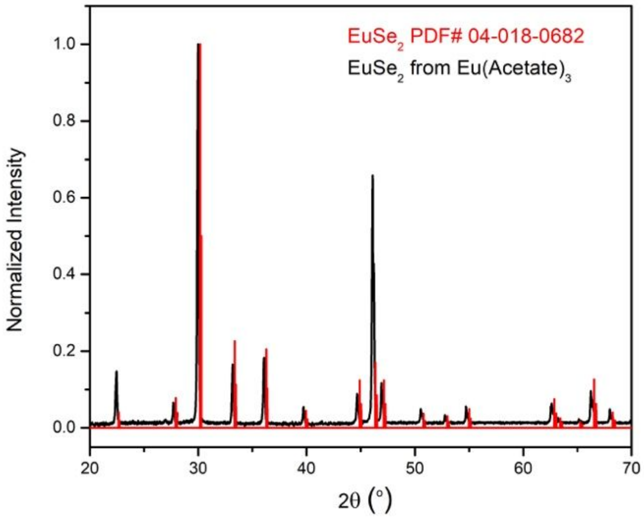

S7. PXRD of $EuSe_2$ from $Eu(O_2CCH_3)_3$.

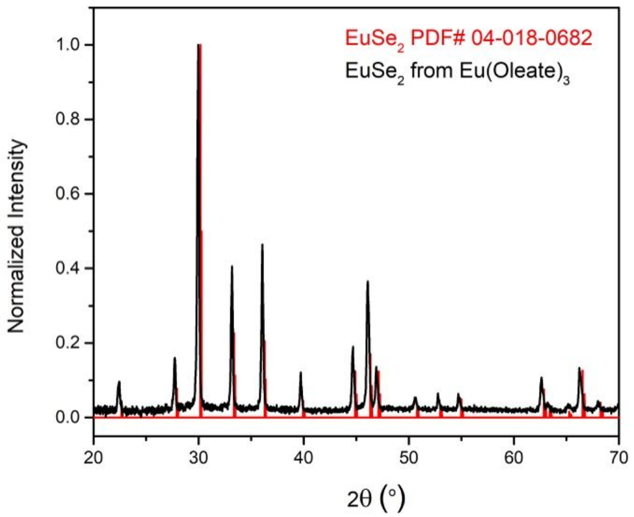

S8. PXRD of $EuSe_2$ from $Eu(oleate)_3$.

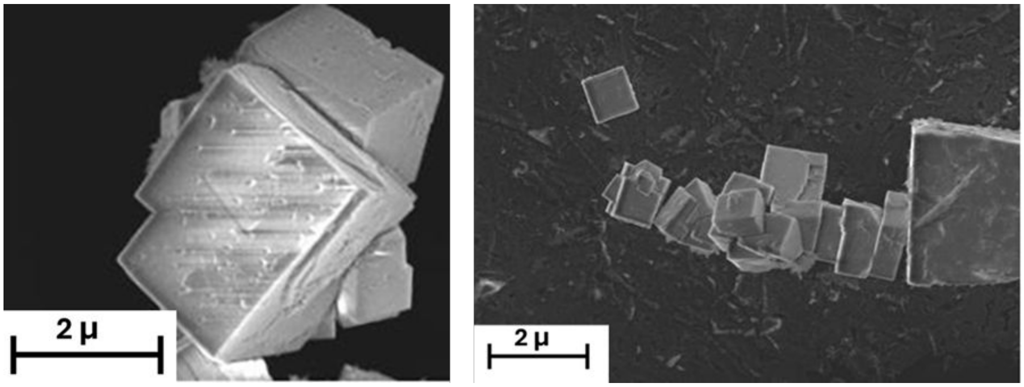

S9. Comparison of nanocrystal morphology using Scanning Electron Microscopy of $EuSe_2$ from $Eu(acetate)_3$ (left) and $Eu(oleate)_3$ (right).

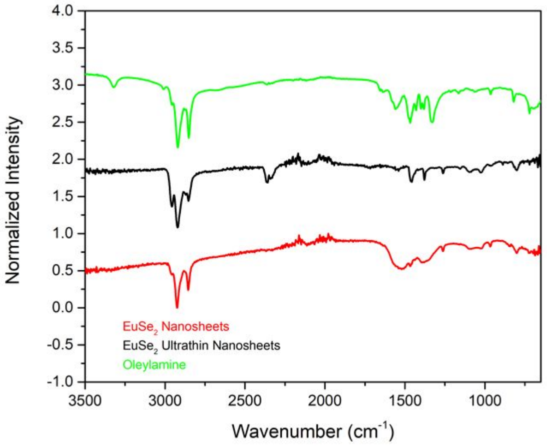

S10. FTIR of the $EuSe_2$ nanosheet and ultra-thin $EuSe_2$.

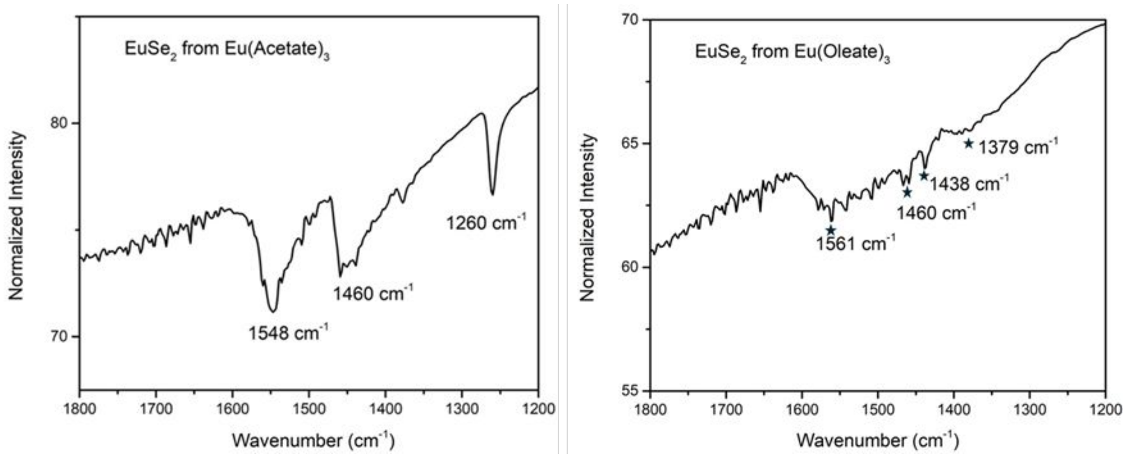

S11. FTIR of the large $EuSe_2$ from acetate (left) and oleate (right) in the carboxylate region.

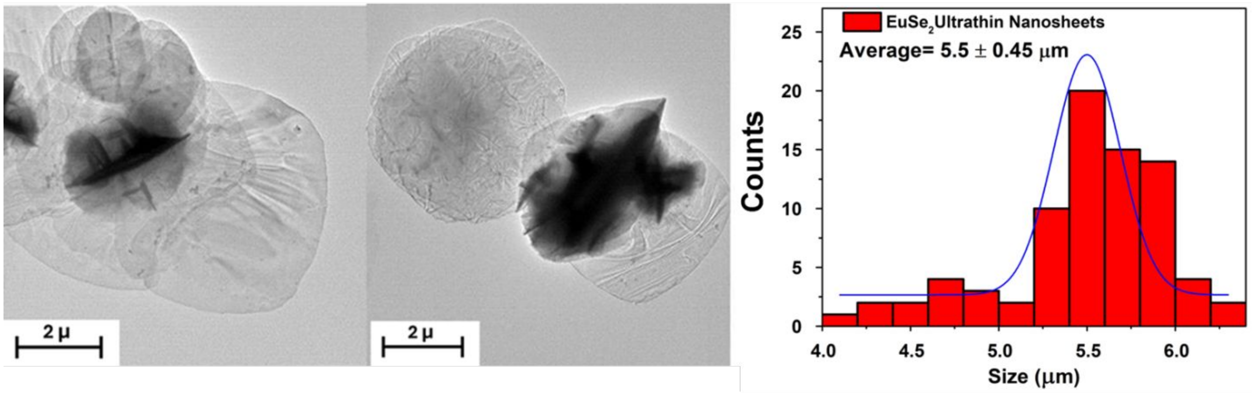

S12. TEM of ultra-thin $EuSe_2$ (from HDA), histogram based on TEM of 92 ultra-thin nanosheets.

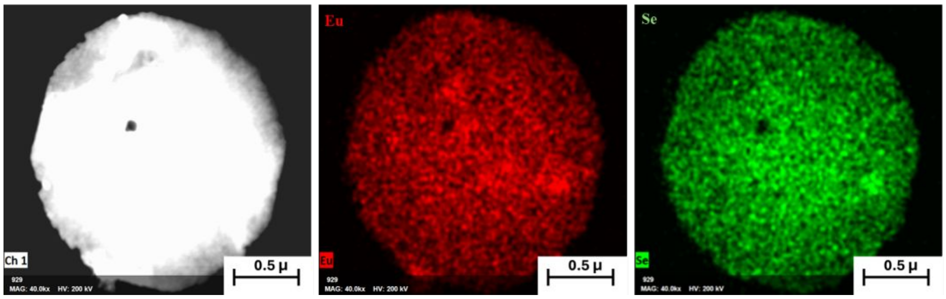

S13. Elemental mapping of ultra-thin $EuSe_2$ nanosheets.

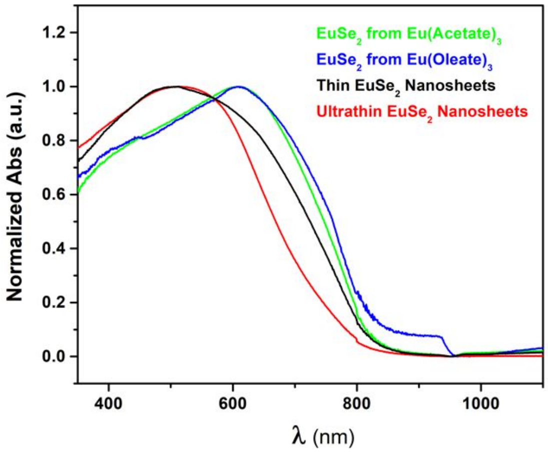

S14. Comparison of UV-visible absorption spectroscopy of $EuSe_2$ from Eu(acetate)$_3$ and Eu(oleate)$_3$ (green and blue) compared from Eu(HMDS)$_3$ from both OLA (black) and HDA (red).

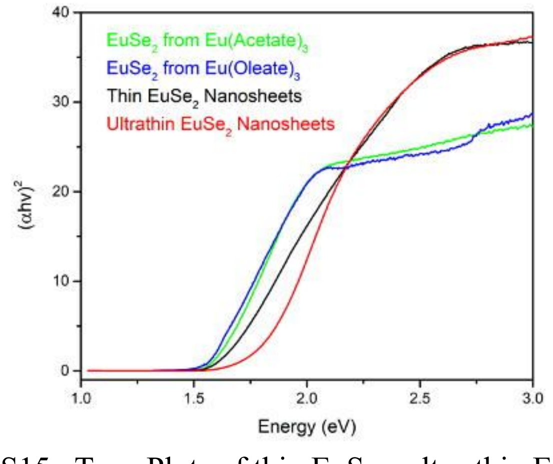

	Direct Bandgap E_g (eV)	R^2
Ultrathin	1.73	0.9878
Thin	1.62	0.99891
Acetate	1.58	0.99604
Oleate	1.55	0.99942

S15. Tauc Plots of thin $EuSe_2$, ultra-thin $EuSe_2$ and $EuSe_2$ from acetate and oleate, assuming direct band gap.

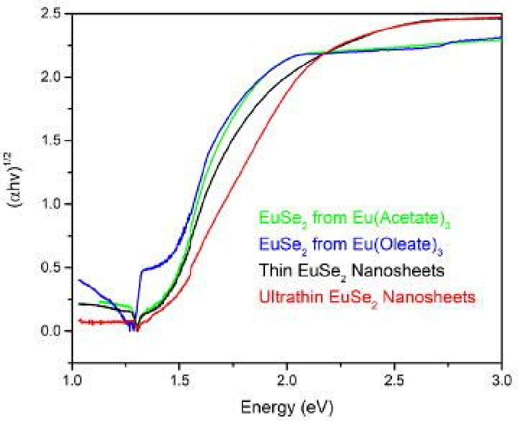

	Indirect Bandgap E_g (eV)	R^2
Ultrathin	1.38	0.99923
Thin	1.38	0.98703
Acetate	1.37	0.97482
Oleate	1.35	0.99042

S16. Tauc Plots of thin $EuSe_2$, ultra-thin $EuSe_2$ and $EuSe_2$ from acetate and oleate, indirect Eg.

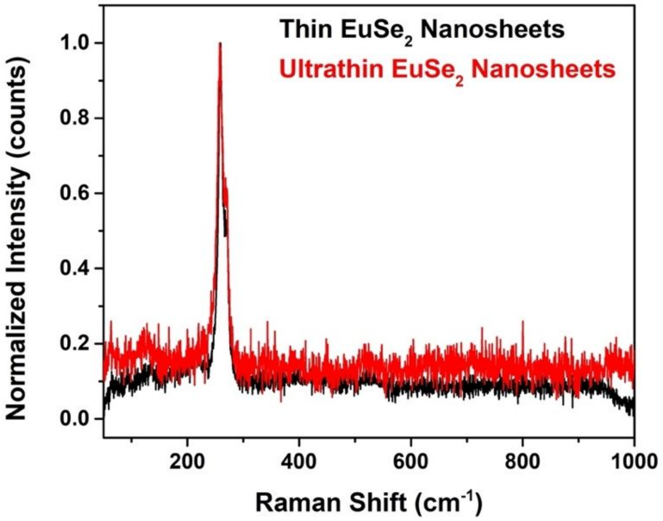

S17. Raman of $EuSe_2$ nanosheets and ultra-thin nanosheets. Peak is at 259-262 cm^{-1} with a shoulder at 270 cm^{-1}.

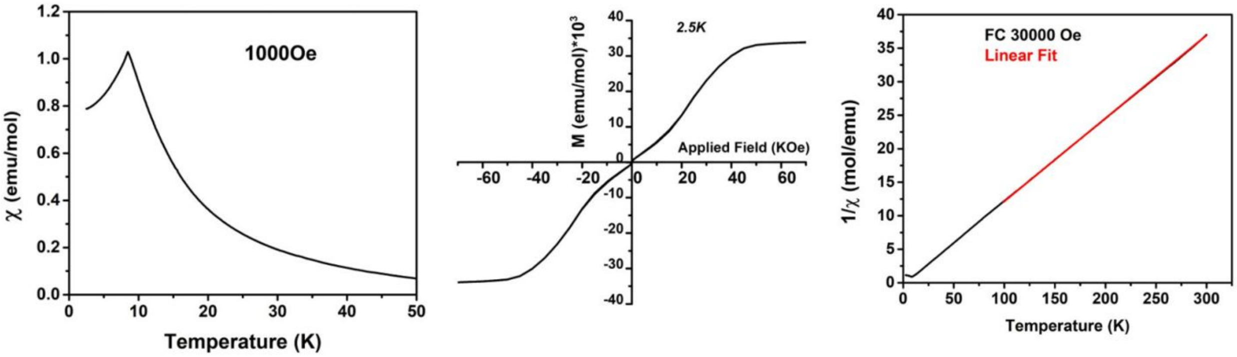

S18. Magnetic data for powders of ultra-thin $EuSe_2$ nanosheets: χ(T) (left), M(H) at 2.5 K (center), Curie Weiss fit (μ_{eff}= 7.91 BM, Θ= 5.8 K) (right).

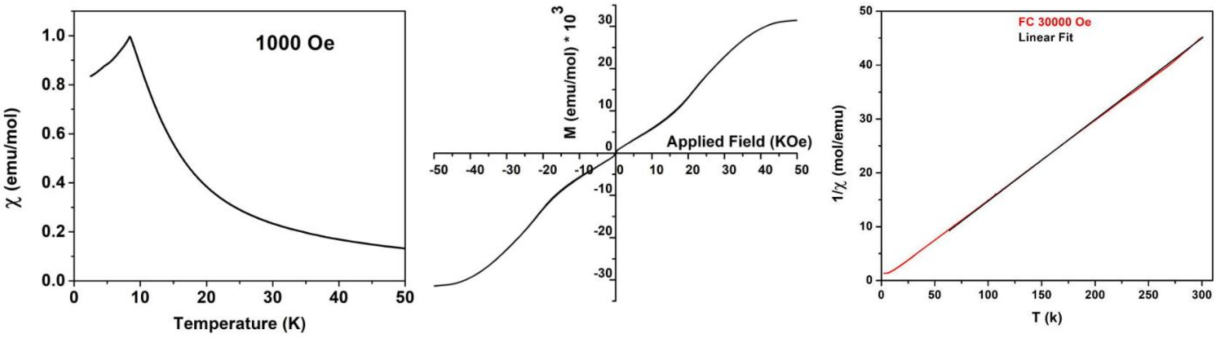

S19. Magnetic data for powders of $EuSe_2$ nanosheets: χ(T) (left), M(H) at 2.5 K (center), Curie Weiss Fit (μ_{eff}= 7.3 BM, Θ= −1.4 K) (right).

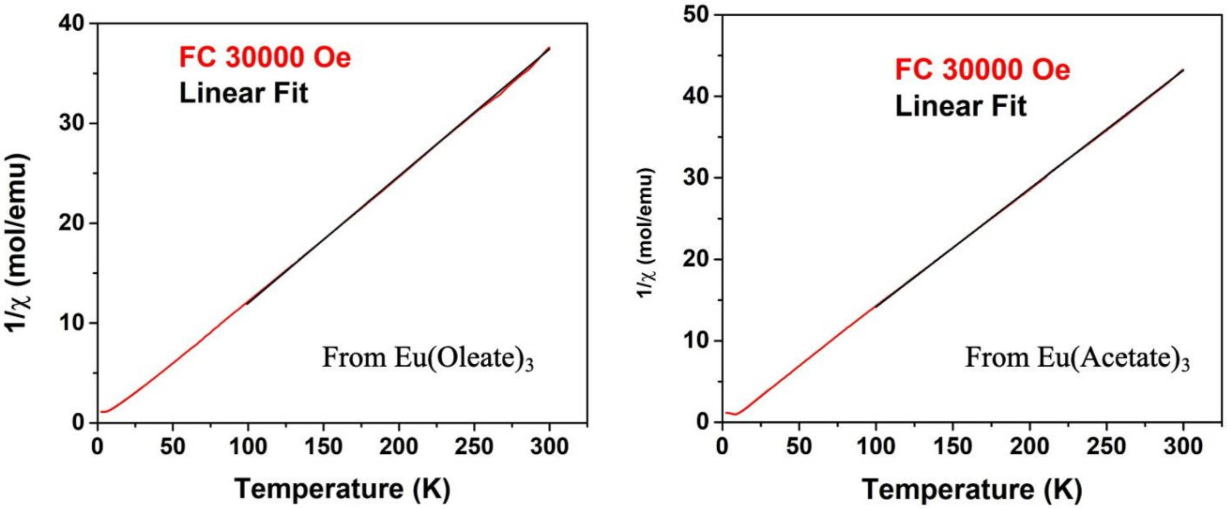

S20. Curie-Weiss plot of the $EuSe_2$ from Eu(acetate)$_3$ and Eu(oleate)$_3$. For the range 100-300 K, the material from Eu(acetate)$_3$, the μ_{eff} was 7.6 BM, and Θ = 1.4 K, while from Eu(oleate)$_3$ the μ_{eff} was 7.9 BM, and Θ = 5.7 K

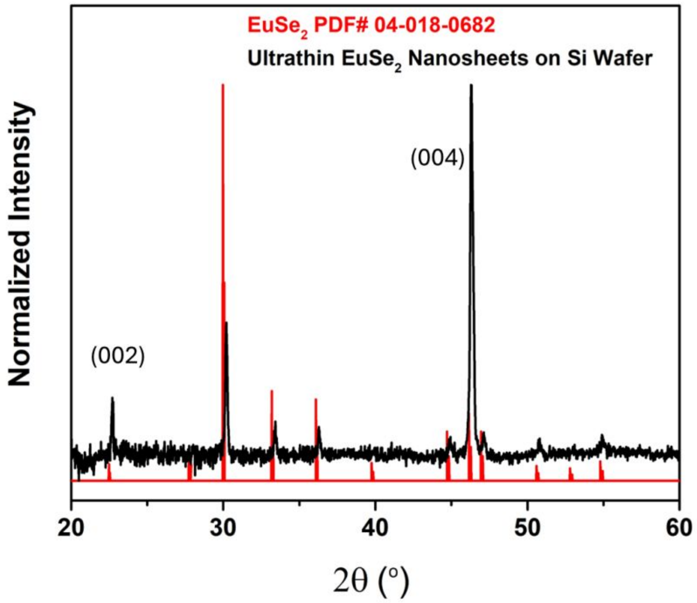

S21. PXRD of $EuSe_2$ ultra-thin nanosheets on Si wafer for thin-film magnetic measurements.
